# The long-acting COX-2 inhibitor mavacoxib (Trocoxil™) has anti-proliferative and pro-apoptotic effects on canine cancer cell lines and cancer stem cells *in vitro*

**DOI:** 10.1186/s12917-014-0184-9

**Published:** 2014-09-05

**Authors:** Lisa Y Pang, Sally A Argyle, Ayako Kamida, Katherine O’Neill Morrison, David J Argyle

**Affiliations:** The Royal (Dick) School of Veterinary Studies and The Roslin Institute, The University of Edinburgh, Easter Bush, Edinburgh, Midlothian EH25 9RG UK

**Keywords:** Mavacoxib, COX-2, Cancer stem cells, Osteosarcoma, Canine

## Abstract

**Background:**

The NSAID mavacoxib (Trocoxcil™) is a recently described selective COX-2 inhibitor used for the management of inflammatory disease in dogs. It has a long plasma half-life, requiring less frequent dosing and supporting increased owner compliance in treating their dogs. Although the use of NSAIDs has been described in cancer treatment in dogs, there are no studies to date that have examined the utility of mavacoxib specifically.

**Results:**

In this study we compared the *in vitro* activity of a short-acting non-selective COX inhibitor (carprofen) with mavacoxib, on cancer cell and cancer stem cell survival. We demonstrate that mavacoxib has a direct cell killing effect on cancer cells, increases apoptosis in cancer cells in a manner that may be independent of caspase activity, and has an inhibitory effect on cell migration. Importantly, we demonstrate that cancer stem cells derived from osteosarcoma cell lines are sensitive to the cytotoxic effect of mavacoxib.

**Conclusions:**

Both NSAIDs can inhibit cancer cell proliferation and induce apoptosis *in vitro.* Importantly, cancer stem cells derived from an osteosarcoma cell line are sensitive to the cytotoxic effect of mavacoxib. Our results suggest that mavacoxib has anti-tumour effects and that this *in vitro* anti-cancer activity warrants further study.

**Electronic supplementary material:**

The online version of this article (doi:10.1186/s12917-014-0184-9) contains supplementary material, which is available to authorized users.

## Background

Cyclooxygenase (COX) is a key enzyme in the synthesis of prostaglandins from arachidonic acid. There are two isoforms of cylooxygenase: COX-1 and COX-2. COX-1 is constitutively expressed in almost all tissues, whereas COX-2 is predominantly induced in response to certain stimuli including growth factors and pro-inflammatory cytokines [1]. Overexpression of COX-2 has been reported in diseases associated with inflammation and various cancers [2,3]. Furthermore, there has been increasing evidence that COX-2 overexpression is associated with tumourigenesis [4], angiogenesis [5], resistance to apoptosis, cancer cell proliferation and metastasis [6,7]. COX-2 protein expression has been associated with a poor prognosis in human breast cancer [8], colon cancer [9], malignant mesothelioma [10], head and neck squamous cell carcinoma [11], and chronic myelogenous leukemia [12].

In recent years the traditional stochastic model of cancer development has been challenged by a new model, which implicates cancer stem cells (CSC) as the subpopulation of cancer cells that drives tumour growth, recurrence and metastasis [13]. CSCs share several characteristics with embryonic and somatic stem cells including self-renewal and differentiation abilities, and represent a small fraction of the cellular population of the tumour [13]. The role of CSCs was initially established in leukaemia, and more recently in solid tumours including melanomas [14,15], glioblastomas [16], and epithelial cancers [17-21]. Increasing evidence has implicated CSCs in tumourigenesis and response to treatment of many tumour types. Significantly, the resistance of these cells to conventional chemotherapeutic regimes suggests that CSCs play a major role in drug resistance and treatment failure [22]. Interestingly, COX-2 has been shown to be upregulated in colon [23] and breast CSCs and constituted part of an eight-gene signature that correlated with breast cancer patient survival [24].

Non-steroidal anti-inflammatory drugs (NSAIDs) inhibit the COX enzyme thereby reducing the production of prostaglandins. NSAIDs are therefore widely used to reduce the clinical signs associated with inflammation. Moreover, increasing evidence from animal models has demonstrated the anti-tumoural and chemopreventative effects that NSAIDs have on several tumours, including intestinal, breast, skin, lung, and bladder tumours [25-28]. In addition, Thun *et al.* (1991) reported that low dose NSAIDs reduced relative risk of colorectal cancer [29], and subsequent studies support increasing evidence that NSAIDs significantly reduce colon polyp formation and recurrence [30,31]. Currently, there are several ongoing human clinical trials utilising NSAIDs as cancer therapeutics [32,33].

In dogs, overexpression of COX-2 and prostaglandin E2 (PGE2) have been identified in a wide variety of cancers, including transitional carcinoma of urinary bladder [34], lymphoma, mammary gland tumours and osteosarcoma [35-37]. As an example, previous studies have shown that COX-2 is not normally expressed in canine bone, but that around 77% of osteosarcomas are positive for COX-2 expression [38]. The suggestion that this makes COX-2 and the prostaglandin PGE2 promising therapeutic targets, is supported by the demonstrable therapeutic benefits of using NSAIDs in tumours that overexpress COX-2, such as prostatic carcinoma [39] and osteosarcoma [40]. Concurrently, use of the NSAID piroxicam is a standard recommendation of treatment for dogs with transitional cell carcinoma. In a pilot study, it was found that 20 percent of dogs with bladder tumours treated with piroxicam alone had a partial or complete response [41].

Mavacoxib (Trocoxil™) is a member of the coxib class of selective COX-2 inhibitors and is approved in the European Union for the treatment of pain and inflammation in canine osteoarthritis, where continuous treatment exceeding 1 month is indicated [42]. Mavacoxib is unique among NSAIDs because its combination of low clearance and relatively large apparent volume of distribution mean that it has a plasma half-life that is much longer than those of other NSAIDs, leading to a much reduced frequency of dosing. The potential benefits of using mavacoxib clinically are therefore high, but to date, there have been no studies evaluating the anti-cancer effects of this drug.

In this study we evaluated the anti-cancer effects of mavacoxib and compared this to another clinically important NSAID, carprofen (a non-selective COX inhibitor). Using a panel of canine cancer cell lines we demonstrate that both drugs can inhibit cancer cell proliferation *in vitro* and we show that both drugs induce apoptosis in cancer cells in a manner that may be independent of caspase activity. Furthermore, mavacoxib, but not carprofen, is cytotoxic to cancer stem cells derived from osteosarcoma cell lines.

## Results

### Mavacoxib (Trocoxil™) inhibits cell proliferation of canine cancer cell lines

**Figure 1:**
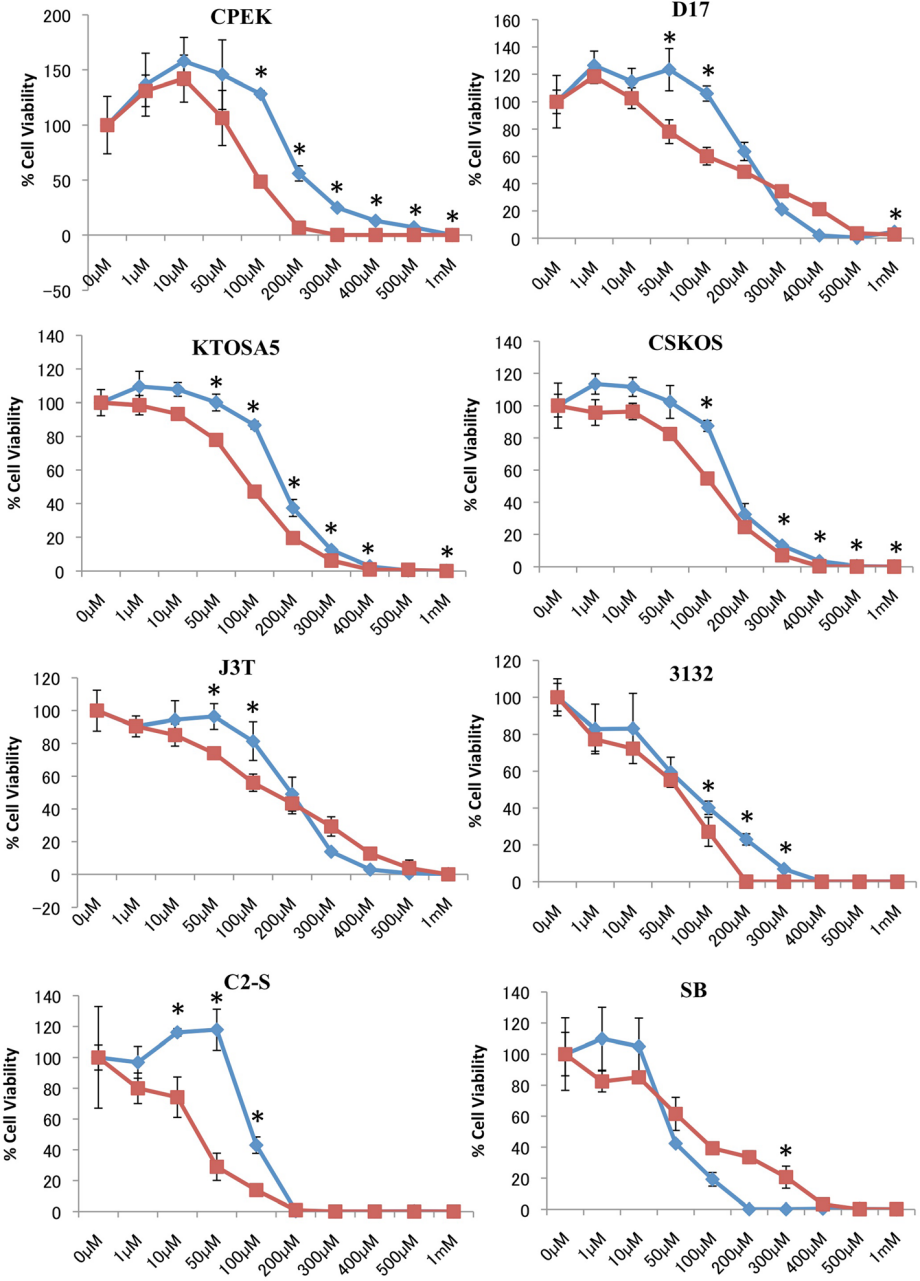
**Mavacoxib inhibits cells proliferation of canine cancer cell lines.** Normal keratinocytes (CPEK); osteosarcoma (D17, KTOSA5, CSKOS); glioma (J3T); lymphoma (3132); mast cell tumour (C2-S); and haemangiosarcoma (SB) cell lines were treated with the indicated doses of either mavacoxib or carprofen and cell viability was assayed 48 hours after treatment. Red line represents mavacoxib. Blue line represents carprofen. *p <0.05 indicate a significant difference between mavacoxib and carprofen at the indicated concentration by a student’s t-test.

A panel of canine cell lines, including CPEK (normal epidermal keratinocyte), D17 (osteosarcoma), KTOSA5 (osteosarcoma), CSKOS (osteosarcoma), J3T (glioma), 3132 (lymphoma), C2-S (mast cell tumour) and SB (hemangiosarcoma) were used to determine the effect of NSAIDs on cell viability (Figure 1). Both carprofen and mavacoxib suppressed cancer cell proliferation effectively in a dose dependent manner, however mavacoxib showed a superior effect in the majority of the cell lines tested (*p* < 0.05), with the exception of SB cells (Figure 1). C2-S showed the greatest sensitivity to mavacoxib with an IC_50_ value of 29.3 μM, whereas 3132 cells showed the greatest sensitivity to carprofen with an IC_50_ value of 63.46 μM (Table 1).

**Table 1:** **Determination of IC**_**50**_ **values for mavacoxib and carprofen in normal keratinocytes (CPEK); osteosarcoma (D17, KTOSA5, CSKOS); glioma (J3T); lymphoma (3132); mast cell tumour (C2-S); and haemangiosarcoma (SB) cell lines**

	**IC**_**50**_	
**Cell type**	**Mavacoxib**	**Carprofen**
CPEK	87.1 μM	226.6 μM
D17	152.7 μM	225.6 μM
KTOSA5	93.4 μM	172.1 μM
CSKOS	107.8 μM	167 μM
J3T	68.4 μM	239.8 μM
3132	36.5 μM	63.5 μM
C2-S	29.3 μM	93.5 μM
SB	63.5 μM	70.4 μM

All measurements were made in triplicate.

### Mavacoxib induces apoptosis in cancer cells and is independent of caspase activation

**Figure 2:**
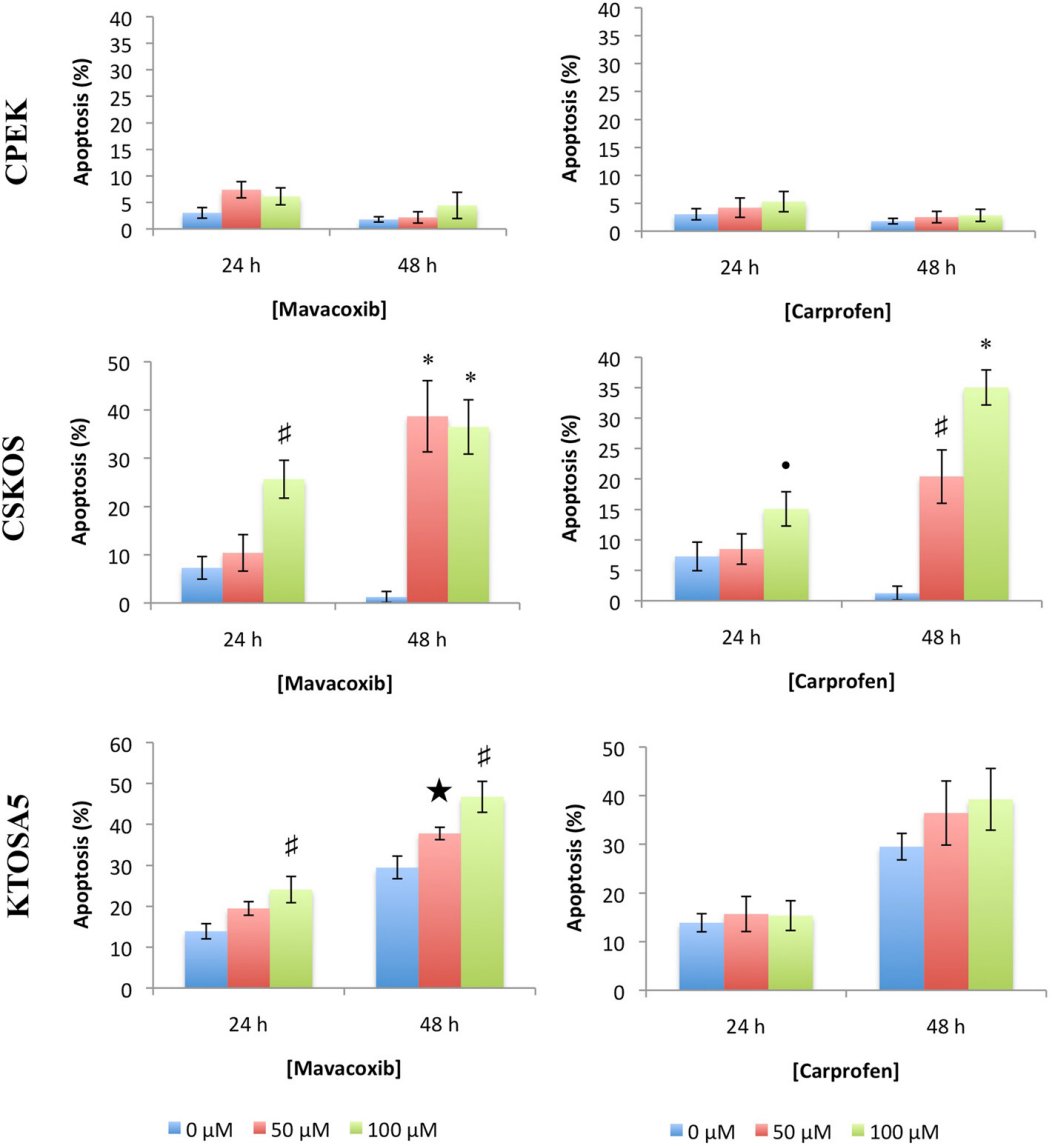
**Effects of NSAIDs on apoptosis.** CPEK, CSKOS, and KTOSA5 cells were treated with the indicated doses of either carprofen or mavacoxib. Annexin V-FITC staining was analysed by FACS 24 hours and 48 hours after treatment. The percentage of Annexin V positive cells was quantified. Ÿ *p* = 0.02, ★ *p* = 0.01, ♯ *p* = 0.002, * *p* < 0.001 by comparing the indicated drug concentration at the indicated time point to the untreated control by a student’s t-test.

To determine the mechanism by which these NSAIDs reduce cell viability, we assayed treated cells for evidence of apoptosis by utilizing Annexin V staining, and explored expression of mediators of apoptosis including caspase activity and levels of Bax and Bcl2. In both KTOSA5 and CSKOS cells, we demonstrated a dose-dependent increase in the percentage of apoptotic cells when either carprofen or mavacoxib were used. Interestingly, both drugs had little effect on non-cancerous CPEK cells, even at high doses of the inhibitor. Both mavacoxib and carprofen had a significant time-dependent and dose-dependent effect on KTOSA5 and CSKOS apoptosis (Figure 2). Generally, the percentage of cells undergoing apoptosis was highest in cells treated with 100 μm NSAIDs for 48 hours, although CSKOS cells were equally effected by 50 μm and 100 μm mavacoxib at 48 hours (Figure 2). To explore the effects on apoptosis further, we investigated the ability of both drugs to cause activation of caspase-2, caspase-8, caspase-9 and caspase-3 following 48 hours of treatment with either mavacoxib or carprofen. We demonstrated no significant difference between the enzyme activity of the initiator caspases -2, -8, -9 and the main effector caspase -3 in cells treated or not treated, suggesting that apoptosis in these cells may be caspase-independent (Additional file 1: Figure S1).

**Figure 3:**
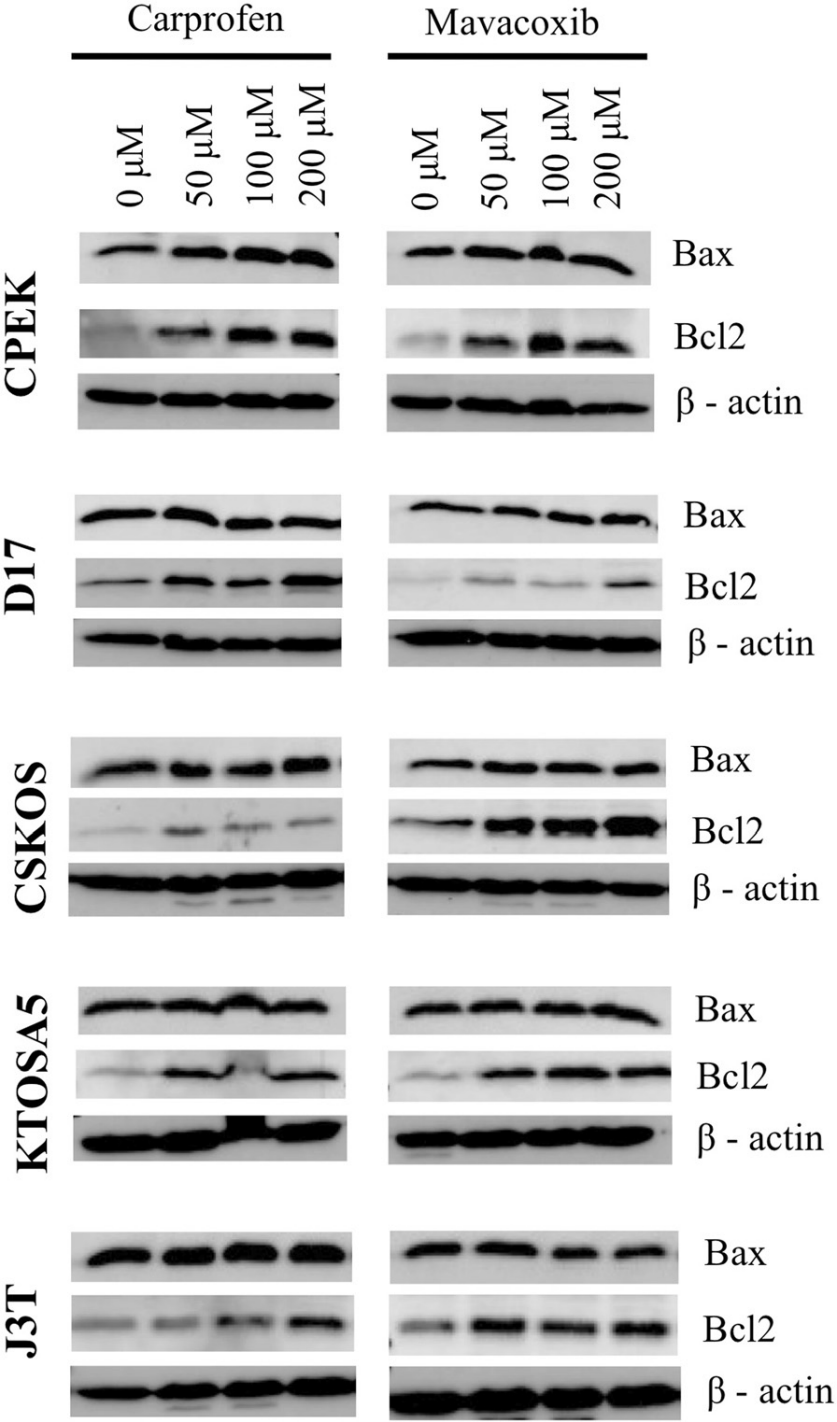
**Carprofen and mavacoxib treatment stimulates Bcl2 and has no effect on basal Bax levels Cells were treated with the indicated dose of either carprofen (C) or mavacoxib (M).** Cells were harvested 48 hours post-treatment.

When we then assayed the treated cells for levels of Bax or Bcl2, we found that treatment stimulated Bcl2 but had no effect on basal Bax levels. Treatment of cells with either carprofen or mavacoxib increased the level of the pro-survival Bcl2 protein, whereas the level of the pro-apoptotic Bax protein was unchanged. These results were consistent in all cell lines tested (Figure 3) and indicate that cell death induced by NSAID treatment does not involve mitochondrial apoptosis.

### Mavacoxib has cell-type dependent effects on cell invasion

**Figure 4:**
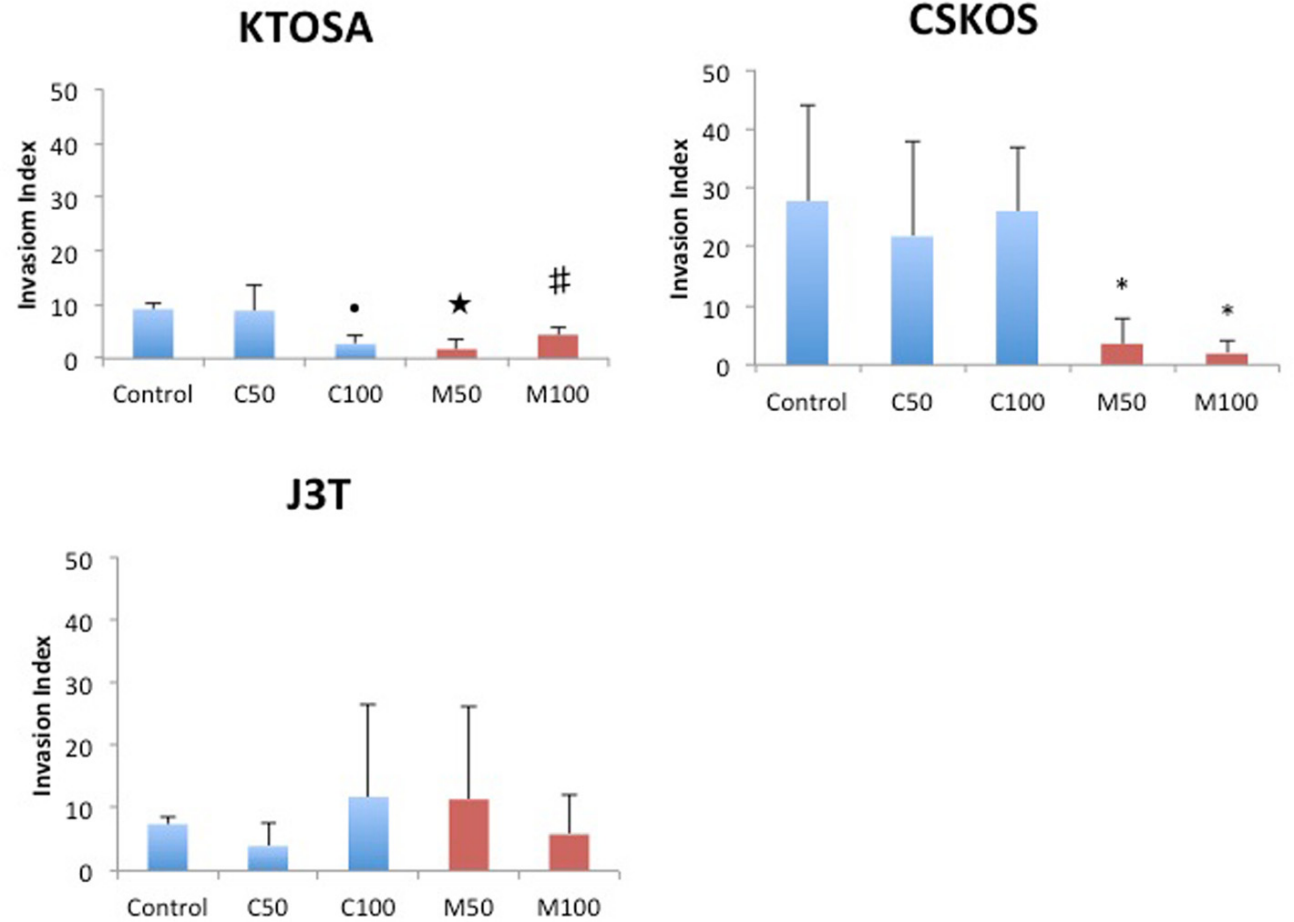
**NSAID treatment has cell type dependent effects on cell invasion.** Cells were treated with the indicated dose of either carprofen (C) or mavacoxib (M). Cells were counted 48 hours post-treatment. ↑ *p* = 0.024 by Mann–Whitney test, ★ *p* = 0.007, ♯ *p* = 0.002, * *p* < 0.001 by a student’s t-test.

The invasion index (%) was calculated for cells treated with either carprofen or mavacoxib. Migrated cells were counted using the transwell assay as described in materials and methods. In the CSKOS cell line, there was no significant difference between the control, untreated cells and cells treated with carprofen. However, in cells treated with mavacoxib there was significant (*p* < 0.001) inhibition of invasion that was dose dependent. KTOSA5 cells demonstrated a similar pattern to CSKOS cells, where the invasion index was unaffected at 50 μM carprofen, but significantly decreased at doses of 100 μM (*p* = 0.024). Treatment with either 50 μM or 100 μM mavacoxib, showed significant inhibition (*p* < 0.02) of invasion (Figure 4). There was no clear inhibition in J3T with either carprofen or mavacoxib treatment (Figure 4).

### Mavacoxib inhibits cell survival of putative cancer stem cells

**Figure 5:**
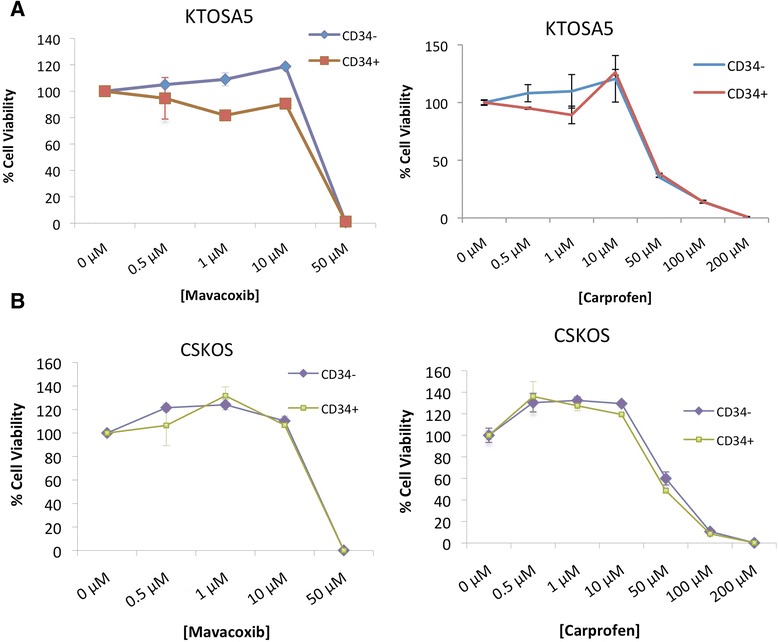
**Sensitivity to NSAIDs is not affected by CD34 status in (A) KTOSA5 cells or (B) CSKOS cells.** Cells were treated with the indicated doses of either mavacoxib or carprofen and cell viability was assayed 48 hours after treatment.

We have previously isolated cancer stem cells from canine osteosarcoma cell lines [43]. As NSAIDs are used as a palliative treatment for dogs with osteosarcoma [44], we wanted to determine the effect of NSAID treatment on the cancer stem cell population of KTOSA5 and CSKOS cell lines. Cells were magnetically sorted for expression of the cancer stem cell marker, CD34 (data unpublished), and assayed for cell viability after treatment with either mavacoxib or carprofen. There was no significant difference between CD34- and CD34+ cells. As before, both mavacoxib and carprofen suppressed cancer cell proliferation effectively in a dose dependent manner, however mavacoxib was more effective at lower concentrations (Figure 5). For example, KTOSA5 CD34+ cells treated with mavacoxib had an IC_50_ value of 36.03 μM, whereas the same cells treated with carprofen had an IC_50_ value of 49.6 (Table 2).

**Table 2:** **Determination of IC**_**50**_ **values for mavacoxib and carprofen in KTOSA5 and CSKOS cells sorted for CD34 protein expression**

		**IC**_**50**_	
**Cell type**	**Drug**	**CD34-**	**CD34+**
KTOSA5	Mavacoxib	38.63 μM	27.37 μM
KTOSA5	Carprofen	42.94 μM	44.87 μM
CSKOS	Mavacoxib	37.62 μM	36.03 μM
CSKOS	Carprofen	55.86 μM	49.6 μM

All measurements were made in triplicate.

**Figure 6:**
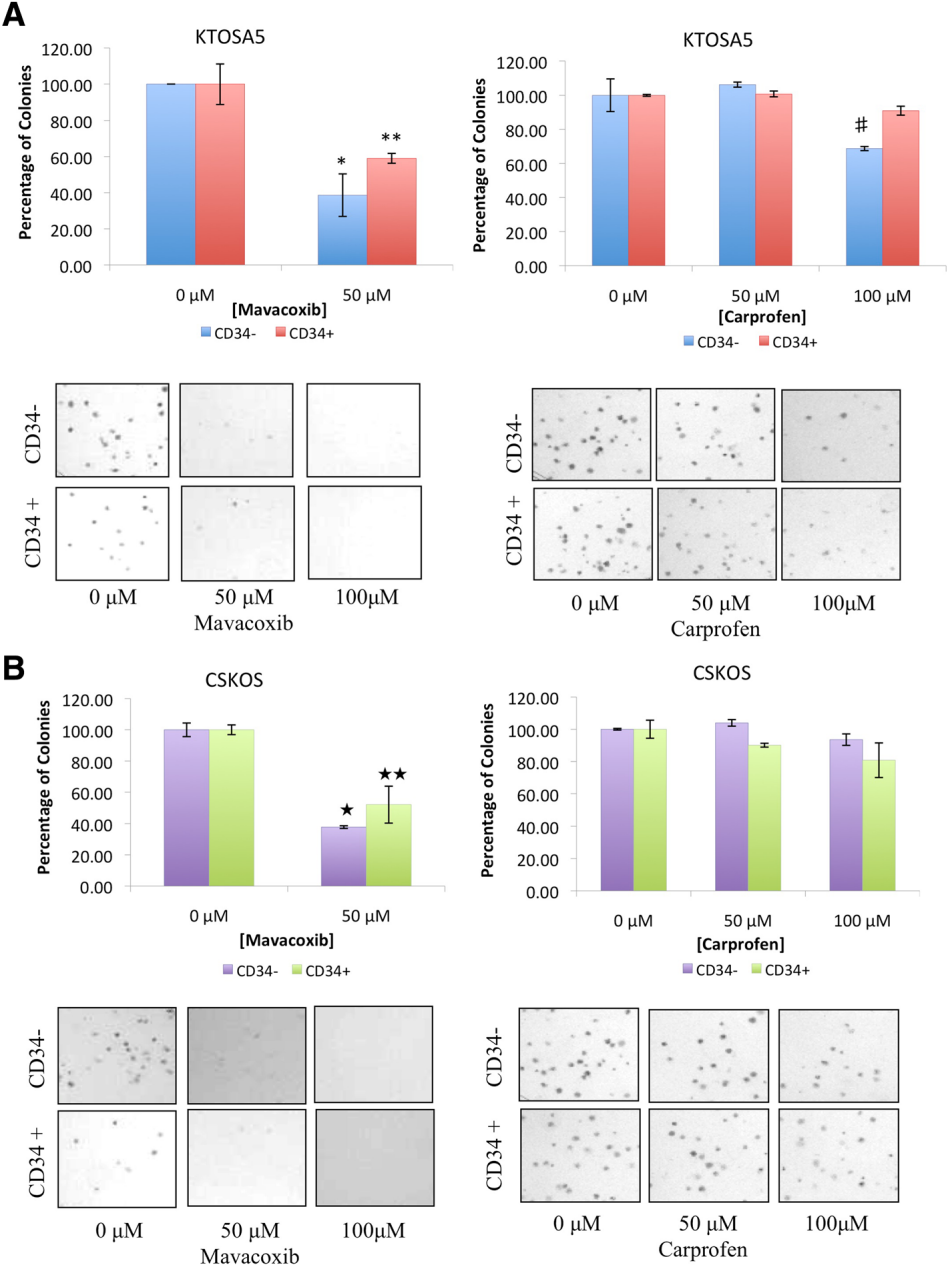
**Mavacoxib is better than carprofen at inhibiting colony forming ability of KTOSA5 cells (A) and CSKOS cells (B) independent of CD34 status.** Cells were treated with the indicated doses of either mavacoxib or carprofen and colony formation was assayed. Number of colonies per plate was counted. Representative images are shown. * *p* = 0.001, ** *p* < 0.001, ♯ *p* = 0.007, ★ *p* < 0.001, ★★ *p* = 0.002 by a student’s t-test.

A colony formation assay was used to determine the effect of NSAID treatment on cell reproductive death. Here, KTOSA5 and CSKOS cells were sorted for CD34 and treated with either mavacoxib or carprofen. As before, both cell lines showed no difference between CD34- and CD34+ cells. In KTOSA5 cells, treatment with 50 μM mavacoxib significantly (p < 0.001) decreased the number of colonies formed compared to untreated cells, and no colonies were formed after treatment with 100 μM mavacoxib. In contrast, treatment with 50 μM carprofen had no effect on the colony forming ability of KTOSA5 cells, and treatment with 100 μM carprofen only inhibited colony formation by approximately 20% (Figure 6A). Similar results were obtained for the CSKOS cell line (Figure 6B). This data indicates that the long-acting COX-2 inhibitor, mavacoxib is a more effective cytotoxic agent than the short-acting non-selective COX inhibitor carprofen.

## Discussion

NSAIDs can be broadly divided into non-selective inhibitors of COX indicating that they inhibit both COX-1 and COX-2 or they can be COX-2 selective. Carprofen falls into the former category inhibiting both COX-1 and COX-2. In addition it is suggested that carprofen may have additional anti-inflammatory mechanisms of action such as inhibition of fatty acid amide hydrolase. In contrast mavacoxib is COX-2 selective, a characteristic of the coxib class of NSAIDs. Meloxicam, although not a coxib, is also considered to be relatively COX-2 selective [45].

When used for its anti-inflammatory properties the prolonged half-life for mavacoxib supports the approved regimen in which doses are separated by 2–4 weeks. In inflammatory conditions, compliance with this regimen is improved when compared to once-daily NSAID treatment [46], and because of the prolonged half-life, a continuous therapeutic response may be more likely in instances when there are problems with dosing, such as a delay in drug administration. Because of the relatively long half-life of mavacoxib, a regimen with a non-constant dosing interval (i.e. 2 weeks between the first two doses but with a maintenance 4 week interval) is used for rapid attainment of steady-state concentrations. With this regimen, plasma mavacoxib concentrations are relatively constant (0.52-1.11 μg/ml). In contrast carprofen has a more typical NSAID half-life in the dog supporting once daily drug administration.

In this current study we have demonstrated that mavacoxib, a COX-2 inhibitor, induced significant cell growth arrest and apoptosis in a panel of canine cancer cell lines, including D17 (osteosarcoma), KTOSA5 (osteosarcoma), CSKOS (osteosarcoma), J3T (glioma), 3132 (lymphoma), C2-S and SB (hemangiosarcoma). In comparison to carprofen, mavacoxib exhibited superior inhibition of cancer cell viability with IC_50_ values 3.5-fold to 1.5-fold higher, depending on the cell line. Interestingly, neither drug induced apoptosis in normal cells (CPEK). Whereas, in the osteosarcoma cell lines tested, both carprofen and mavacoxib induced a dose-dependent increase in the percentage of apoptotic cells after 24 and 48 hours, indicating that cancer cells may be more sensitive to the killing effect of COX inhibition compared to normal cells.

Apoptosis is a conserved cell death mechanism essential for normal development and tissue homeostasis. Classically, apoptotic cell death is characterized by caspase activation, down-regulation of Bcl2 and up-regulation of pro-apoptotic Bax [47]. Here, we show that apoptosis induced by either mavacoxib or carprofen is independent of the initiator caspases 2, 8 and 9, and the effector caspase 3. We also show that all the cell lines tested have a high basal level of Bax, which is not induced by increasing doses of either mavacoxib or carprofen. Concurrently, there is a dose-dependent increase in levels of anti-apoptotic Bcl2. Previous studies have shown that both apoptotic and anti-apoptotic factors are up-regulated in human tumours, and this leads to an impairment of the apoptotic signaling pathway and can confer resistance to apoptosis [48]. However, we observed this pattern in normal cells that have a functioning apoptotic pathway. We also confirmed that cell death was due to apoptosis and not necrosis. Caspase-independent and Bax-independent cell death has previously been characterised [49]. Further investigation is required to determine if mavacoxib induces caspase-independent cell death in canine cancer cell lines. In human studies of osteosarcoma and gastric cancer cell lines treated with meloxicam and SC236 (a potent COX-2 selective inhibitor) respectively, no induction of Bax protein was demonstrated [35,50]. Naruse *et al*. (2006) discussed that the COX-2 selective NSAID meloxicam may affect a Bax-independent apoptotic pathway, which is yet to be elucidated [35].

We also demonstrated that in the osteosarcoma cell lines, KTOSA5 and CSKOS, high doses of mavacoxib are better at inhibiting invasiveness compared to carprofen. In the J3T glioma cell line there was no difference in invasiveness compared to control, indicating that COX-2 may have cell type specific effects. The mechanism of this inhibition requires further investigation, but it suggests that mavacoxib may have potential therapeutic benefits. Previous studies have demonstrated that COX-2 derived PGE2 activates CD44 and MMP-2, and in turn stimulates invasiveness of lung cancer cells *in vitro* [51]. In addition, COX-2 inhibition of the human osteosarcoma cell line, MG-63, by meloxicam can also inhibit invasiveness [35]. The role of COX-2 in the tumour microenvironment should also be considered; Williams *et al.* (2000) showed that the tumours grown in COX-2-null mice had decreased growth, as well as vascular density, compared to wild type mice [52], suggesting that both host cells and tumour cells are affected by COX-2 expression levels.

To fully determine the anti-proliferative effect of COX-2 inhibition on canine cancer, it is fundamental to study the effect of COX-2 inhibition on the cancer stem cell population. Cancer stem cells are a small subpopulation of cells that can influence initiation, recurrence and chemoresistance of a tumour [13]. As we have previously isolated cancer stem cells from canine osteosarcoma cell lines [43] and NSAIDs are used as a palliative treatment for dogs with osteosarcoma, we used the canine osteosarcoma cell lines, to look at the effect of COX-2 inhibition with mavacoxib. Cancer stem cells isolated from these cell lines are inherently more resistant to the cytotoxic effect of the anthracycline antibiotic, doxorubicin (data unpublished). Here, we show that there is no significant difference between cancer cells and putative cancer stem cells. This is significant as it shows that cancer stem cells are not resistant to COX-2 inhibition. As before, mavacoxib is more effective at killing cells at a lower concentration than carprofen.

We also determined the long-term effect of COX-2 inhibition on replicative cell death of both cancer and cancer stem cells. Significantly, mavacoxib dramatically decreased the colony forming ability in a dose-dependent manner in both cell populations, whereas carprofen only had a modest effect. Mavacoxib is a long-acting COX-2 inhibitor with a relatively long plasma half-life compared to carprofen, and this may account for the difference in colony forming ability. As we have shown here, mavacoxib is more effective than carprofen as a cytotoxic agent against both cancer cells and cancer stem cells.

Mavacoxib is closely related to the human drug, celecoxib. Previous studies have shown that putative cancer stem cells isolated from human glioma cell lines express constitutively high levels of COX-2 protein, which are positively correlated with radioresistance. Treatment with celecoxib enhanced radiosensitivity of glioma stem cells and suppressed the expression of angiogenic and stemness-related genes [53]. To date, the molecular mechanism by which the COX-2 inhibitors, celecoxib and mavacoxib, exert their anti-tumour effect on the cancer stem cell population is yet to be elucidated. Further studies should be conducted to determine if mavacoxib also has synergistic effects with other chemotherapeutic agents. The data presented here demonstrates that the anti-proliferative effects of mavacoxib are effective on a range of canine cancer cell types and that the therapeutic potential of mavacoxib, either as a single agent or as a multimodal therapy, should be further investigated.

## Conclusions

Our study provides the first evidence that mavacoxib is an effective anti-tumour agent. Mavacoxib can inhibit cell proliferation and invasiveness better than carprofen. Importantly, mavacoxib can inhibit cancer stem cell survival in an *in vitro* osteosarcoma model. Therefore, mavacoxib could be a potential candidate for the treatment of canine cancer and further studies of its clinical use are now required.

## Methods

### Cell culture

The KTOSA5 cell line was derived from an osteosarcoma affecting the right hind limb of an 8-year-old, chemotherapy naïve, female entire Rottweiler (approved by the University of Edinburgh Veterinary Ethical Review Committee). KTOSA5 (osteosarcoma), CSKOS (osteosarcoma), J3T (glioma) cell lines were grown in Dulbecco’s modified Eagle’s medium (DMEM) (Invitrogen, Paisley, UK) supplemented with 10% fetal bovine serum and 100 μg/ml streptomycin (Invitrogen, Paisley, UK). D17 (osteosarcoma), C2-S (mast cell tumour) and SB (hemangiosarcoma) cells were grown in DMEM-GlutaMAX™ (Invitrogen) supplemented with 10% fetal bovine serum and 100 μg/ml streptomycin. 3132 (lymphoma) cells were grown in RPMI medium 1640 (Invitrogen) supplemented with 10% fetal bovine serum and 100 μg/ml streptomycin. CPEK (normal epidermal keratinocyte) were cultured in CnT-09 epidermal keratinocyte medium (CELLnTEC, Bern, Switzerland). All cell cultures were maintained at 37°C in a humidified CO_2_ incubator.

### Magnetic cell sorting

Cells were labelled with CD34 microbeads and sorted using the Miltenyi Biotec CD34 cell isolation kit according to the manufacturer’s protocol (Miltenyi Biotec, Surrey, UK). Briefly, cells were resuspended in 300 μl PBS solution (pH 7.2, 0.5% BSA, 2 mM EDTA) per 10^8^ cells. Then blocking reagent FcR (100 μl/10^8^ cells; Miltenyi Biotec, Surrey, UK) and CD34 microbeads (100 μl/10^8^ cells) were added and mixed at 4°C for 30 minutes with rotation. Cells were washed in 20× volume with PBS solution. The pellet was resuspended in 500 μl PBS solution and added to a pre-washed magnetic separation (LS) column on the magnetic holder. The column was washed four times and the collected cells were designated as the negative fraction. The column was removed from the magnetic holder and the positive fraction was collected.

### Cell viability assay

To determine cell viability, CellTiter-Glo® Luminescent Cell Viability Assay kit (Promega, Madison, WI, USA) was used according to manufacturer’s instructions. Briefly, 500 cells were seeded per well in a 96-well plate, in triplicate. Cells were incubated for 24 h at 37°C, 5% CO_2_. Drugs were applied at a range of concentrations (0 μM – 5 mM) and incubated for 48 h. The plate was equilibrated at room temperature for 30 min and 100 μl of CellTiter-Glo® reagent was added to each well. Luminescence was recorded by a luminometor (Viktor3, PerkinElmer, Massachusetts, USA). IC_50_ values were calculated from three replicates using GraphPad Prism version 5 software (GraphPad Software Inc., San Diego, CA).

### Colony formation assay

Cells were trypsinised into single cells and seeded at 500 cells/10 cm plate. The cells were incubated with appropriate doses of either mavacoxib or carprofen whilst in suspension. Plates were incubated at 37°C in a humidified CO_2_ incubator until colonies were visible. Growth media was changed once a week. The colonies were fixed by incubating with ice-cold methanol for 5 minutes at room temperature. Colonies were stained with Giemsa stain (Invitrogen, Paisley, UK) according to the manufacturer’s instructions and the number of colonies were counted.

### Caspase profiling assay

Activation of caspase-2, caspase-8, caspase-9 and caspase-3 on indicated drug treatments was determined using a caspase profiling plate (Clontech lab., CA, USA) according to the manufacturer’s instructions. Briefly, cells were grown until 60-70% confluent, then treated with either carprofen or mavacoxib (50 μM and 100 μM), and incubated for 48 h at 37°C, 5% CO_2_. Untreated cells were also prepared as a control for each cell line. After treatment, 2 × 10^5^ cells were prepared per well, lysed in cold 1× Cell Lysis Buffer (50 μl/2 × 10^5^ cells) and incubated for 10 min on ice. The plates were preincubated with 50 μl of 2× Reaction buffer (mixed with 0.1 M DTT) to each well for 5 min at 37°C, 5% CO_2_. Cell lysates were transferred to each well and incubated for 2 h at 37°C, 5% CO_2_. Caspase activities were read using a fluorescent plate reader (excitation: 380 nm, emission: 460 nm) (Viktor3, PerkinElmer, Massachusetts, USA).

### Annexin V assay

The BD FITC Annexin V Apoptosis Detection kit (BD PharMingen, San Diego, CA) was used to determine the effect of carprofen or mavacoxib treatment on the level of apoptosis. The Annexin V analysis allows monitoring of changes in necrosis, early apoptosis and late apoptosis. The assay was performed according to the manufacturer’s instructions. Briefly, cells were grown until 60-70% confluent and treated with either carprofen or mavacoxib at final concentrations of 50 μM and 100 μM. Untreated cells were used as controls for each cell line. Cells were treated for 24 h and 48 h at 37°C, 5% CO_2_. Subsequently, cells were washed twice with cold PBS, trypsinized and resuspended in 1 ml of 0.1% BSA containing PBS. 1 × 10^5^ cells/ml and were then stained with Annexin V FITC and propidium iodide for 15 min in the dark. The CyAn ADP analyser (Beckman Coulter Inc. CA, USA) was used and the data was analyzed using Summit v4.3 software.

### Migration and invasion assay

To evaluate the effect of carprofen and mavacoxib on the metastatic potential of the cell lines, motility and invasiveness were analysed using a transwell migration and invasion assay. CPEK, KTOSA5, CSKOS, D17 and J3T cells were grown to 60-70% confluency and then treated with carprofen or mavacoxib at concentrations of 50 μM and 100 μM. Cell lines were incubated for 48 h at 37°C, 5% CO_2_. Subsequently, cells were detached and re-suspended in FBS-free medium. For the migration assay, 2.5 × 10^4^ cells in 500 μl volume were plated on the top chamber with a non-coated polycarbonate membrane with 8.0 μm pore size (BD Bioscience, Bedford, MA, USA) in duplicate. Medium with 10% FBS was added in the lower chamber as a chemoattractant. After incubation for 24 h, the cells that migrated to the lower surface of the polycarbonate membrane were fixed with methanol for 1 min, followed by Giemsa stain for 15 min. The cells which did not migrate through the pores, were mechanically removed by a cotton swab moistened with the medium. The mean number of migrated cells was counted in 5 random fields of each membrane. The invasion assay was performed as for the previously described migration assay, except that the transwell inserts consist of Matrigel-coated polycarbonate membranes with 8.0 μm pore size. Matrigel mimics the basement membrane *in vitro* and provides a barrier to non-invasive cells. Data is expressed as the percent invasion calculated by the following formula; % Invasion = (Mean number of cells invading/Mean number of cells migrating) × 100.

### Protein detection

Cells were lysed in urea lysis buffer (7 M urea, 0.1 M DTT, 0.05% Triton X-100, 25 mM NaCl, 20 mM Hepes pH 7.5). Equal amounts of protein were separated by SDS polyacrylamide gel electrophoresis (SDS PAGE), transferred to Hybond-C nitrocellulose membrane (Amersham Pharmacia Biotech, Buckinghamshire, UK) and hybridised to an appropriate primary antibody and HRP-conjugated secondary antibody for subsequent detection by ECL. Primary antibody against β-actin was purchased from Abcam (Cambridge, UK). Antibodies against Bax and Bcl2 were purchased from Santa Cruz Biotechnology (CA, USA). The secondary antibodies, HRP-conjugated rabbit anti-mouse IgG and swine anti-rabbit IgG, were obtained from DakoCytomation (Glostrup, Denmark).

### Statistical analysis

Data were expressed as mean ± SD. Statistical analysis was performed with Minitab® statistical software (PA, USA) using analysis of variance and student’s t test or Mann–Whitney test. The criterion for significance was p < 0.05 for all comparisons.

## Additional file

Additional file 1: Figure S1. NSAID treatment has cell-type dependent effects on caspase activity. Cells were treated with either 50 μM or 100 μM carprofen (C) or mavacoxib (M) for 48 hours. Caspase activity was determined using the apoalert caspase assay kit (Calbiochem). Blue bars represents carprofen treatment. Red bar represents mavacoxib treatment.

## References

[1] Greenhough A, Smartt HJ, Moore AE, Roberts HR, Williams AC, Paraskeva C, Kaidi A (2009). The COX-2/PGE2 pathway: key roles in the hallmarks of cancer and adaptation to the tumour microenvironment. Carcinogenesis.

[2] Rizzo MT (2011). Cyclooxygenase-2 in oncogenesis. Clin Chim Acta.

[3] Xu Z, Choudhary S, Voznesensky O, Mehrotra M, Woodard M, Hansen M, Herschman H, Pilbeam C (2006). Overexpression of COX-2 in human osteosarcoma cells decreases proliferation and increases apoptosis. Cancer Res.

[4] Liu CH, Chang SH, Narko K, Trifan OC, Wu MT, Smith E, Haudenschild C, Lane TF, Hla T (2001). Overexpression of cyclooxygenase-2 is sufficient to induce tumorigenesis in transgenic mice. J Biol Chem.

[5] Tsujii M, Kawano S, Tsuji S, Sawaoka H, Hori M, DuBois RN (1998). Cyclooxygenase regulates angiogenesis induced by colon cancer cells. Cell.

[6] Tsujii M, DuBois RN (1995). Alterations in cellular adhesion and apoptosis in epithelial cells overexpressing prostaglandin endoperoxide synthase 2. Cell.

[7] Hiraga T, Myoui A, Choi ME, Yoshikawa H, Yoneda T (2006). Stimulation of cyclooxygenase-2 expression by bone-derived transforming growth factor-beta enhances bone metastases in breast cancer. Cancer Res.

[8] Costa C, Soares R, Reis-Filho JS, Leitao D, Amendoeira I, Schmitt FC (2002). Cyclo-oxygenase 2 expression is associated with angiogenesis and lymph node metastasis in human breast cancer. J Clin Pathol.

[9] Peng L, Zhou Y, Wang Y, Mou H, Zhao Q (2013). Prognostic significance of COX-2 immunohistochemical expression in colorectal cancer: a meta-analysis of the literature. PLoS One.

[10] Edwards JG, Faux SP, Plummer SM, Abrams KR, Walker RA, Waller DA, O’Byrne KJ (2002). Cyclooxygenase-2 expression is a novel prognostic factor in malignant mesothelioma. Clin Cancer Res.

[11] Gallo O, Masini E, Bianchi B, Bruschini L, Paglierani M, Franchi A (2002). Prognostic significance of cyclooxygenase-2 pathway and angiogenesis in head and neck squamous cell carcinoma. Hum Pathol.

[12] Giles FJ, Kantarjian HM, Bekele BN, Cortes JE, Faderl S, Thomas DA, Manshouri T, Rogers A, Keating MJ, Talpaz M, O'Brien S, Albitar M (2002). Bone marrow cyclooxygenase-2 levels are elevated in chronic-phase chronic myeloid leukaemia and are associated with reduced survival. Br J Haematol.

[13] Pang LY, Argyle DJ (2009). Using naturally occurring tumours in dogs and cats to study telomerase and cancer stem cell biology. Biochim Biophys Acta.

[14] Fang D, Nguyen TK, Leishear K, Finko R, Kulp AN, Hotz S, Van Belle PA, Xu X, Elder DE, Herlyn M (2005). A tumorigenic subpopulation with stem cell properties in melanomas. Cancer Res.

[15] Schatton T, Murphy GF, Frank NY, Yamaura K, Waaga-Gasser AM, Gasser M, Zhan Q, Jordan S, Duncan LM, Weishaupt C, Fuhlbrigge RC, Kupper TS, Sayegh MH, Frank MH (2008). Identification of cells initiating human melanomas. Nature.

[16] Singh SK, Clarke ID, Terasaki M, Bonn VE, Hawkins C, Squire J, Dirks PB (2003). Identification of a cancer stem cell in human brain tumors. Cancer Res.

[17] Al-Hajj M, Wicha MS, Benito-Hernandez A, Morrison SJ, Clarke MF (2003). Prospective identification of tumorigenic breast cancer cells. Proc Natl Acad Sci U S A.

[18] Bapat SA, Mali AM, Koppikar CB, Kurrey NK (2005). Stem and progenitor-like cells contribute to the aggressive behavior of human epithelial ovarian cancer. Cancer Res.

[19] Collins AT, Berry PA, Hyde C, Stower MJ, Maitland NJ (2005). Prospective identification of tumorigenic prostate cancer stem cells. Cancer Res.

[20] Ricci-Vitiani L, Lombardi DG, Pilozzi E, Biffoni M, Todaro M, Peschle C, De Maria R (2007). Identification and expansion of human colon-cancer-initiating cells. Nature.

[21] Eramo A, Lotti F, Sette G, Pilozzi E, Biffoni M, Di Virgilio A, Conticello C, Ruco L, Peschle C, De Maria R (2008). Identification and expansion of the tumorigenic lung cancer stem cell population. Cell Death Differ.

[22] Pang LY, Argyle D (2010). Cancer stem cells and telomerase as potential biomarkers in veterinary oncology. Vet J.

[23] Deng Y, Su Q, Mo J, Fu X, Zhang Y, Lin EH (2013). Celecoxib downregulates CD133 expression through inhibition of the Wnt signaling pathway in colon cancer cells. Cancer Invest.

[24] Kanojia D, Zhou W, Zhang J, Jie C, Lo PK, Wang Q, Chen H (2012). Proteomic profiling of cancer stem cells derived from primary tumors of HER2/Neu transgenic mice. Proteomics.

[25] Ferrandina G, Lauriola L, Distefano MG, Zannoni GF, Gessi M, Legge F, Maggiano N, Mancuso S, Capelli A, Scambia G, Ranelletti FO (2002). Increased cyclooxygenase-2 expression is associated with chemotherapy resistance and poor survival in cervical cancer patients. J Clin Oncol.

[26] Cha YI, DuBois RN (2007). NSAIDs and cancer prevention: targets downstream of COX-2. Annu Rev Med.

[27] Karamouzis MV, Papavassiliou AG (2004). COX-2 inhibition in cancer therapeutics: a field of controversy or a magic bullet?. Expert Opin Investig Drugs.

[28] Gendy AS, Lipskar A, Glick RD, Steinberg BM, Edelman M, Soffer SZ (2011). Selective inhibition of cyclooxygenase-2 suppresses metastatic disease without affecting primary tumor growth in a murine model of Ewing sarcoma. J Pediatr Surg.

[29] Thun MJ, Namboodiri MM, Heath CW (1991). Aspirin use and reduced risk of fatal colon cancer. N Engl J Med.

[30] Steinbach G, Lynch PM, Phillips RK, Wallace MH, Hawk E, Gordon GB, Wakabayashi N, Saunders B, Shen Y, Fujimura T, Su LK, Levin B, Godio L, Patterson S, Rodriguez-Bigas MA, Jester SL, King KL, Schumacher M, Abbruzzese J, DuBois RN, Hittelman WN, Zimmerman S, Sherman JW, Kelloff G (2000). The effect of celecoxib, a cyclooxygenase-2 inhibitor, in familial adenomatous polyposis. N Engl J Med.

[31] Langley RE, Burdett S, Tierney JF, Cafferty F, Parmar MK, Venning G (2011). Aspirin and cancer: has aspirin been overlooked as an adjuvant therapy?. Br J Cancer.

[32] Almhanna K, El-Rayes B, Sethi S, Dyson G, Heilbrun L, Philip PA, Sarkar F (2012). Association between COX-2 expression and effectiveness of COX-2 inhibitors in a phase II trial in patients with metastatic colorectal adenocarcinoma. Anticancer Res.

[33] Rizzo MT (2011). Cyclooxygenase-2 in oncogenesis. Clin Chim Acta.

[34] Knapp DW, Richardson RC, Chan TC, Bottoms GD, Widmer WR, DeNicola DB, Teclaw R, Bonney PL, Kuczek T (1994). Piroxicam therapy in 34 dogs with transitional cell carcinoma of the urinary bladder. J Vet Intern Med.

[35] Naruse T, Nishida Y, Hosono K, Ishiguro N (2006). Meloxicam inhibits osteosarcoma growth, invasiveness and metastasis by COX-2-dependent and independent routes. Carcinogenesis.

[36] Mohammed SI, Coffman K, Glickman NW, Hayek MG, Waters DJ, Schlittler D, DeNicola DB, Knapp DW (2001). Prostaglandin E2 concentrations in naturally occurring canine cancer. Prostaglandins Leukot Essent Fatty Acids.

[37] Brunelle M, Sartin EA, Wolfe LG, Sirois J, Dore M (2006). Cyclooxygenase-2 expression in normal and neoplastic canine mammary cell lines. Vet Pathol.

[38] Mullins MN, Lana SE, Dernell WS, Ogilvie GK, Withrow SJ, Ehrhart EJ (2004). Cyclooxygenase-2 expression in canine appendicular osteosarcomas. J Vet Intern Med.

[39] Sorenmo KU, Goldschmidt MH, Shofer FS, Goldkamp C, Ferracone J (2004). Evaluation of cyclooxygenase-1 and cyclooxygenase-2 expression and the effect of cyclooxygenase inhibitors in canine prostatic carcinoma. Vet Comp Oncol.

[40] Wolfesberger B, Walter I, Hoelzl C, Thalhammer JG, Egerbacher M (2006). Antineoplastic effect of the cyclooxygenase inhibitor meloxicam on canine osteosarcoma cells. Res Vet Sci.

[41] Mutsaers AJ, Widmer WR, Knapp DW (2003). Canine transitional cell carcinoma. J Vet Intern Med.

[42] Cox SR, Lesman SP, Boucher JF, Krautmann MJ, Hummel BD, Savides M, Marsh S, Fielder A, Stegemann MR (2010). The pharmacokinetics of mavacoxib, a long-acting COX-2 inhibitor, in young adult laboratory dogs. J Vet Pharmacol Ther.

[43] Wilson H, Huelsmeyer M, Chun R, Young KM, Friedrichs K, Argyle DJ (2008). Isolation and characterisation of cancer stem cells from canine osteosarcoma. Vet J.

[44] Fan TM, de Lorimier LP, O’Dell-Anderson K, Lacoste HI, Charney SC (2007). Single-agent pamidronate for palliative therapy of canine appendicular osteosarcoma bone pain. J Vet Intern Med.

[45] Bertolacci L, Romeo E, Veronesi M, Magotti P, Albani C, Dionisi M, Lambruschini C, Scarpelli R, Cavalli A, De Vivo M, Piomelli D, Garau G (2013). A binding site for nonsteroidal anti-inflammatory drugs in fatty acid amide hydrolase. J Am Chem Soc.

[46] Payne-Johnson M (2009). Efficacy and safety of mavacoxib in the treatment of pain and inflammation associated with degenerative joint disease in dogs presented as veterinary patients. 11th International Congress of the European Association for Veterinary Pharmacology and Toxicology.

[47] Meier P, Vousden KH (2007). Lucifer’s labyrinth–ten years of path finding in cell death. Mol Cell.

[48] Yang L, Cao Z, Yan H, Wood WC (2003). Coexistence of high levels of apoptotic signaling and inhibitor of apoptosis proteins in human tumor cells: implication for cancer specific therapy. Cancer Res.

[49] Kroemer G, Martin SJ (2005). Caspase-independent cell death. Nat Med.

[50] Fan XM, Jiang XH, Gu Q, Ching YP, He H, Xia HH, Lin MC, Chan AO, Yuen MF, Kung HF, Wong BC (2006). Inhibition of Akt/PKB by a COX-2 inhibitor induces apoptosis in gastric cancer cells. Digestion.

[51] Dohadwala M, Batra RK, Luo J, Lin Y, Krysan K, Pold M, Sharma S, Dubinett SM (2002). Autocrine/paracrine prostaglandin E2 production by non-small cell lung cancer cells regulates matrix metalloproteinase-2 and CD44 in cyclooxygenase-2-dependent invasion. J Biol Chem.

[52] Williams CS, Tsujii M, Reese J, Dey SK, DuBois RN (2000). Host cyclooxygenase-2 modulates carcinoma growth. J Clin Invest.

[53] Ma HI, Chiou SH, Hueng DY, Tai LK, Huang PI, Kao CL, Chen YW, Sytwu HK (2011). Celecoxib and radioresistant glioblastoma-derived CD133+ cells: improvement in radiotherapeutic effects. Laboratory investigation. J Neurosurg.

