# Supplementary figures and images for: The long-acting COX-2 inhibitor mavacoxib (Trocoxil™) has anti-proliferative and pro-apoptotic effects on canine cancer cell lines and cancer stem cells in vitro
Source: BMC Vet Res. 2014 Sep 5;10:184. doi: 10.1186/s12917-014-0184-9 (PMC4172958; doi:10.1186/s12917-014-0184-9)

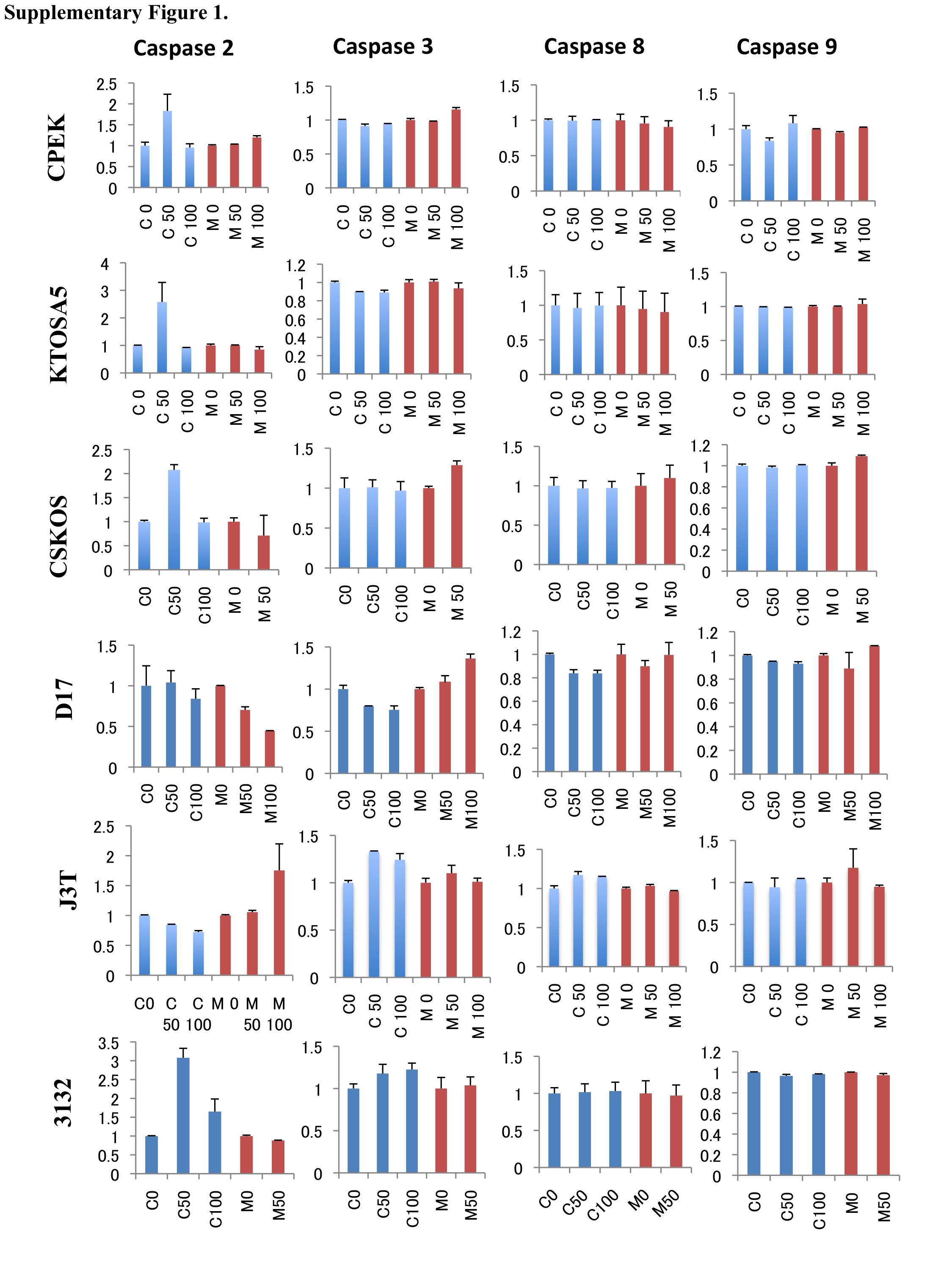

Supplement: Additional file 1: Figure S1. — NSAID treatment has cell-type dependent effects on caspase activity. Cells were treated with either 50 μM or 100 μM carprofen (C) or mavacoxib (M) for 48 hours. Caspase activity was determined using the apoalert caspase assay kit (Calbiochem). Blue bars represents carprofen treatment. Red bar represents mavacoxib treatment. [file 12917_2014_184_MOESM1_ESM.jpeg]
